# Preclinical to Clinical Translation of Studies of Transcranial Direct-Current Stimulation in the Treatment of Epilepsy: A Systematic Review

**DOI:** 10.3389/fnins.2018.00189

**Published:** 2018-03-22

**Authors:** Gabriela G. Regner, Patrícia Pereira, Douglas T. Leffa, Carla de Oliveira, Rafael Vercelino, Felipe Fregni, Iraci L. S. Torres

**Affiliations:** ^1^Laboratory of Neuropharmacology and Preclinical Toxicology, Institute of Basic Health Sciences, Universidade Federal do Rio Grande do Sul, Porto Alegre, Brazil; ^2^Laboratory of Pain Pharmacology and Neuromodulation, Preclinical Studies - Pharmacology Department, Institute of Basic Health Sciences, Universidade Federal do Rio Grande do Sul, Porto Alegre, Brazil; ^3^Postgraduate Program in Biological Sciences, Pharmacology and Therapeutics, Institute of Basic Health Sciences, Universidade Federal Rio Grande do Sul, Porto Alegre, Brazil; ^4^Postgraduate Program in Medical Sciences, School of Medicine Universidade Federal Rio Grande do Sul, Porto Alegre, Brazil; ^5^Centro Universitário FADERGS, Health and Wellness School Laureate International Universities, Porto Alegre, Brazil; ^6^Laboratory of Neuromodulation, Department of Physical Medicine & Rehabilitation, Spaulding Rehabilitation Hospital and Massachusetts General Hospital, Harvard University, Boston, MA, United States

**Keywords:** animal models, clinical trials, epilepsy, non-invasive brain stimulation, transcranial direct current stimulation

## Abstract

Epilepsy is a chronic brain syndrome characterized by recurrent seizures resulting from excessive neuronal discharges. Despite the development of various new antiepileptic drugs, many patients are refractory to treatment and report side effects. Non-invasive methods of brain stimulation, such as transcranial direct current stimulation (tDCS), have been tested as alternative approaches to directly modulate the excitability of epileptogenic neural circuits. Although some pilot and initial clinical studies have shown positive results, there is still uncertainty regarding the next steps of investigation in this field. Therefore, we reviewed preclinical and clinical studies using the following framework: (1) preclinical studies that have been successfully translated to clinical studies, (2) preclinical studies that have failed to be translated to clinical studies, and (3) clinical findings that were not previously tested in preclinical studies. We searched PubMed, Web of Science, Embase, and SciELO (2002–2017) using the keywords “tDCS,” “epilepsy,” “clinical trials,” and “animal models.” Our initial search resulted in 64 articles. After applying inclusion and exclusion criteria, we screened 17 full-text articles to extract findings about the efficacy of tDCS, with respect to the therapeutic framework used and the resulting reduction in seizures and epileptiform patterns. We found that few preclinical findings have been translated into clinical research (number of sessions and effects on seizure frequency) and that most findings have not been tested clinically (effects of tDCS on status epilepticus and absence epilepsy, neuroprotective effects in the hippocampus, and combined use with specific medications). Finally, considering that clinical studies on tDCS have been conducted for several epileptic syndromes, most were not previously tested in preclinical studies (Rasmussen's encephalitis, drug resistant epilepsy, and hippocampal sclerosis-induced epilepsy). Overall, most studies report positive findings. However, it is important to underscore that a successful preclinical study may not indicate success in a clinical study, considering the differences highlighted herein. Although most studies report significant findings, there are still important insights from preclinical work that must be tested clinically. Understanding these factors may improve the evidence for the potential use of this technique as a clinical tool in the treatment of epilepsy.

## Introduction

Techniques involving stimulation of the central nervous system have been extensively studied in recent years. These techniques have been shown to improve symptoms in a range of neurological disorders. Both invasive and non-invasive brain stimulation techniques have been described. Non-invasive techniques can be divided into transcranial magnetic stimulation (TMS), transcranial alternating current stimulation, and transcranial direct current stimulation (tDCS) (Woods et al., 2016).

**Figure 1:**
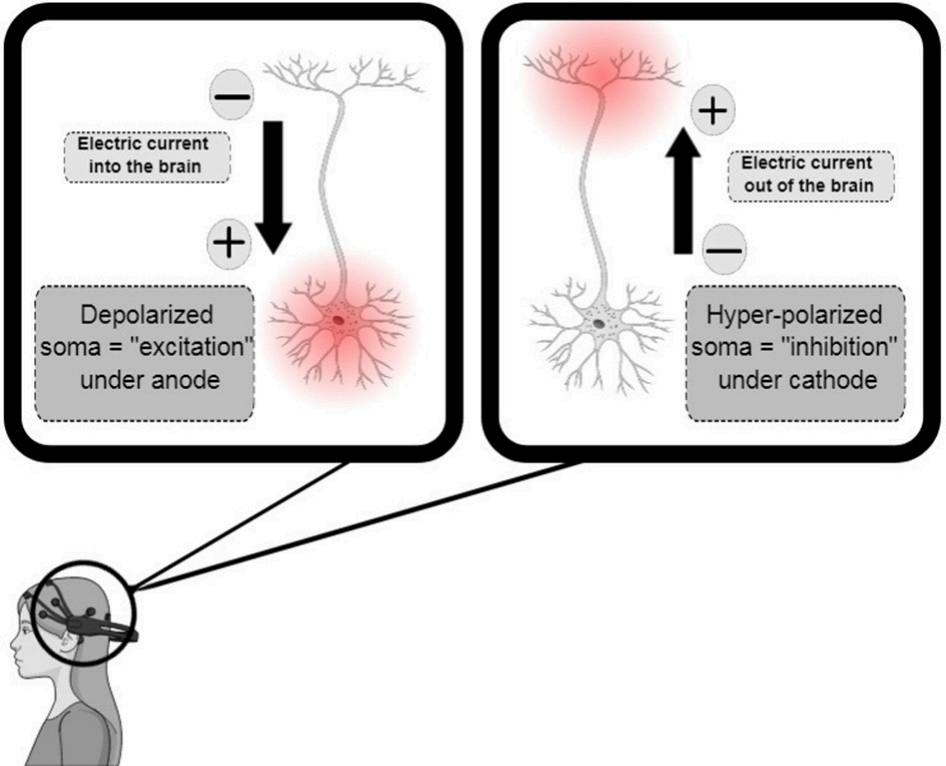
Transcranial direct-current stimulation design, demonstrating anodal, and cathodal stimuli with consequent depolarization (increase in excitability) and hyperpolarization (decrease in excitability), respectively. Adapted from Jackson et al. (2016).

**Figure 2:**
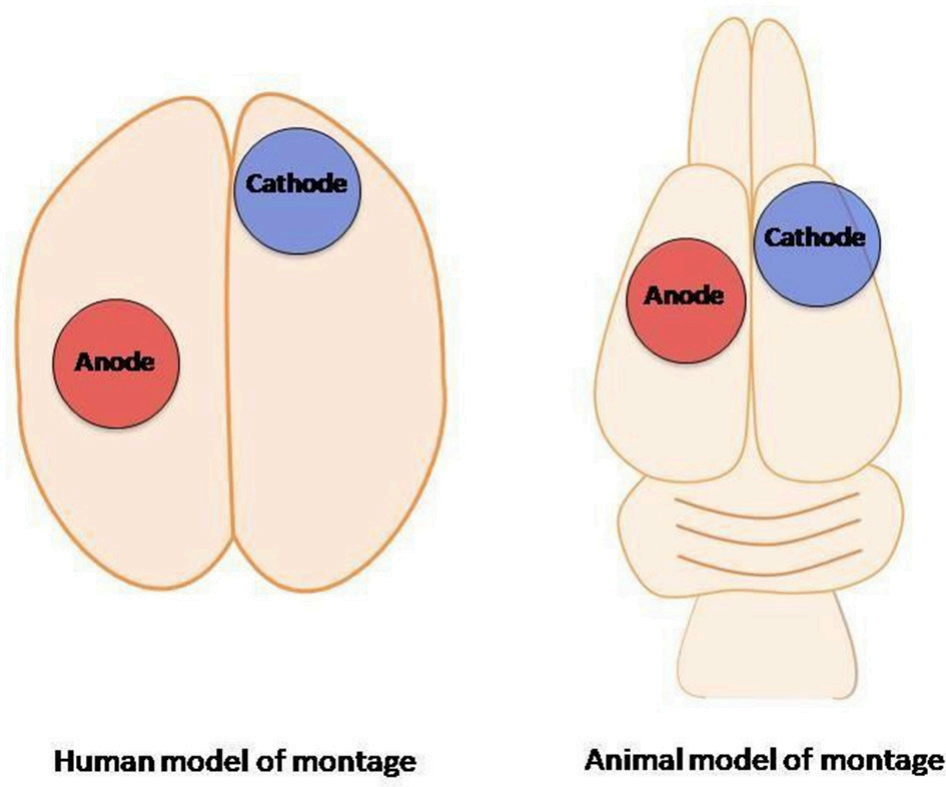
Transcranial direct-current stimulation: human montage vs. transcranial direct-current stimulation animal model (from author).

tDCS relies on the modification of the neuronal resting membrane potential to induce changes in cortical excitability. This technique consists on applying a weak, direct, constant, and low intensity electric current over the scalp using two electrodes: an anode and a cathode (Gomez Palacio Schjetnan et al., 2013). The electrodes are arranged in different assemblies, creating a flow of low-level continuous electrical current targeting a specific region of the cerebral cortex. Anodal stimulation induces depolarization of the neuronal membrane, and therefore facilitates neuronal firing. In contrast, cathodal stimulation has the opposite effect, hyperpolarizing the neuronal membrane (Figure 1; Jackson et al., 2016). tDCS is applied at intensities ranging from 0.5–2 mA across saline-soaked electrodes placed on the human or animal scalp (Figure 2) [from author].

tDCS has been shown to improve symptoms in patients with depression, stroke, focal dystonia, migraine, chronic pain, and epilepsy (Liebetanz et al., 2006a). Epilepsy represents a chronic brain syndrome of diverse etiology, characterized by recurrent seizures resulting from excessive neuronal discharge (Chindo et al., 2014). This syndrome affects ~0.5–1% of the world's population (Dhir et al., 2005), and is associated with a variety of clinical symptoms such as impaired consciousness, movement and sensation (Chindo et al., 2014). Epileptical discharges are generated in response to a loss of balance between excitatory and inhibitory connections, resulting in tonic depolarization of brain circuits (McCormick and Contreras, 2001). The pathophysiology of epilepsy includes hyperactivity of excitatory glutamatergic transmission, and a deficit of inhibitory signaling, mainly resulting from insufficient neurotransmission mediated by γ-aminobutyric acid A receptors (GABA_AR)_ (Lason et al., 2013).

Despite the diversity of new antiepileptic drugs, a large proportion of individuals suffering from epilepsy is refractory to pharmacology treatment (Loscher, 2002), and/or reports side effects which hinder the use of drugs. Epidemiological data indicate that 20–40% of patients with newly diagnosed epilepsy become refractory to treatment (Loscher and Schmidt, 2004; French, 2007). For this reason, there is a continuous search for new therapeutic strategies, from which tDCS has emerged as a possible alternative. In this study, we aimed to summarize the evidence concerning the effects of tDCS in epilepsy in both clinical and preclinical studies. We used a framework to provide insight into the translation rate of preclinical into clinical studies. In addition, we attempted to determine important areas for clinical testing, to verify if these results are complementary, and to identify possible limitations of these studies.

## Methods

### Search strategy

**Figure 3:**
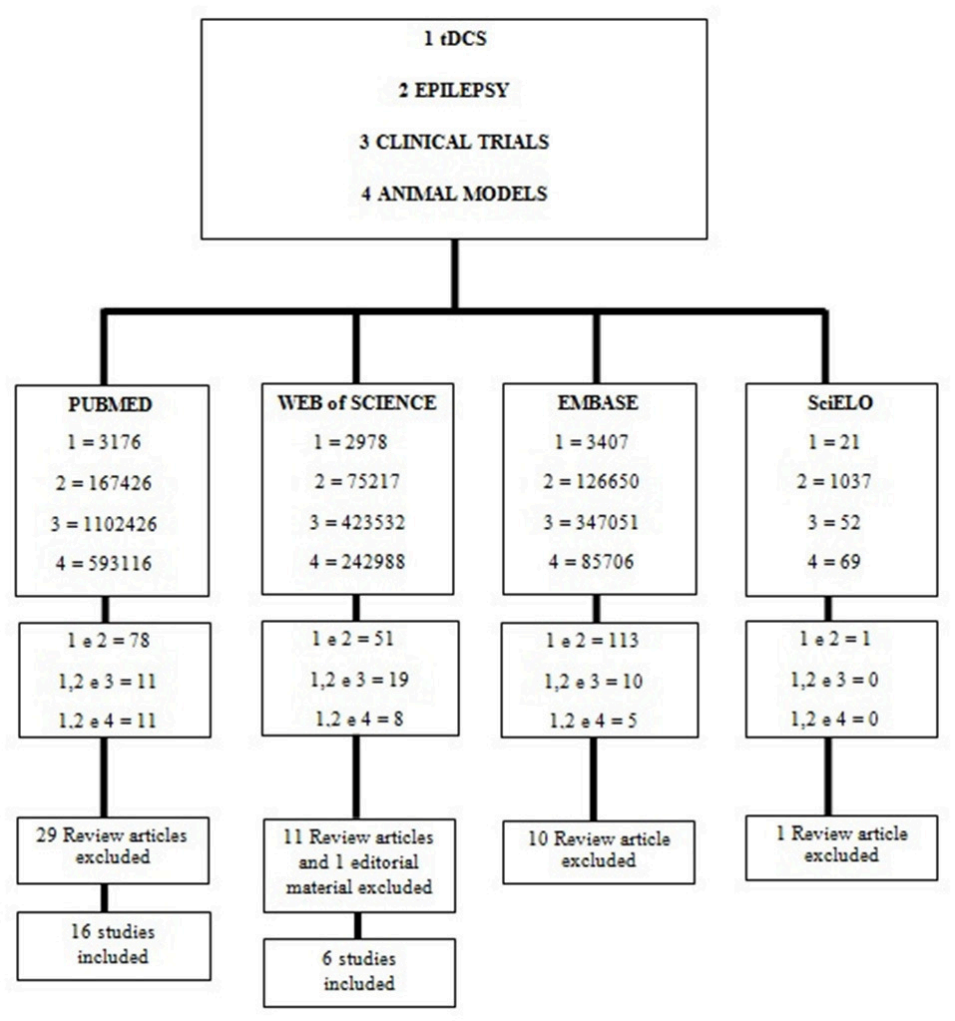
Flowchart of the query on transcranial direct-current stimulation in major databases.

This systematic review was based on a literature search using PubMed, Web of Science, Embase and SciELO. The keyword “tDCS” was used in combination with other keywords such as “epilepsy,” “clinical trials,” and “animal models.” The term “AND” was used in each combination (Figure 3). In addition, the reference sections of the studies that met our inclusion criteria were manually screened for relevant publications.

### Inclusion and exclusion criteria

Studies had to meet the following criteria: (1) publication in English between 2002 and 2017, (2) report original research, and (3) report case reports. Exclusion criterion were: (1) lack of original data (e.g. review articles, editorial material, articles reporting duplicate data); (2) articles addressing only effects of other brain stimulation techniques, such as alternating electrical current stimulation, or TMS.

### Data extraction and outcomes

Two investigators (GGR and CO) extracted data from the full articles independently. Any disagreements were resolved by a third investigator (PP). However, we summarized the results in a narrative format. The primary outcome of our study was seizure suppression (SS). Relevant articles reporting the outcome of interest were identified, and standardized tables were utilized to extract the following variables: experiment, total sessions, interval between sessions, number of individuals (N), montage, contact area of electrodes, sham group and results/insights. The primary author collected the name of the authors, titles and study design. When further information about the study was needed, the authors were contacted by email.

### Risk of bias assessment

Risk of bias was assessed by two reviewers (GGR and CO), for each included preclinical study, using SYRCLE's Risk of Bias tool for animal studies (Hooijmans et al., 2014). We extracted study characteristics related to construct and external validity (Henderson et al., 2013). For construct validity, we included sex, species and strain, type of epilepsy model, total treatment sessions and the interval between them, and the use of any other intervention. For clinical studies, we ranked each of the following as having a high, low, or unclear risk of bias when included allocation concealment, blinding of participants, personnel and outcome assessors, incomplete outcome data, and selective outcome reporting (Higgins and Green, 2011). Since the primary outcome included only the tDCS effectiveness, not harmful effects, results may be biased.

### Framework of translation from preclinical studies to clinical studies

In order to analyze the results for this review, we used a framework to understand whether preclinical findings were translated into clinical studies, and whether or not this translational research confirmed preclinical findings. Therefore, we created three main categories: (1) preclinical studies that have been successfully translated in clinical studies, (2) preclinical studies that have failed to translate in clinical studies, (3) clinical findings that were not tested previously in preclinical studies.

## Results

The final search identified 64 studies. After applying the inclusion and exclusion criteria, we included 17 articles with different types of designs (5 preclinical/12 clinical) for full-text analysis. We screened the articles according to the main outcome, the suppression of seizures, and summarized the results separately for basic (Table 1) and clinical (Table 2) research. Interestingly, the results obtained from the search in PubMed and Web of Science were the same. We summarized our findings using the framework discussed above.

**Table 1:** Summary of tDCS and animal models of epilepsy.

**Author (year)**	**Title**	**Type of article**	**Experiment**	**Total sessions**	**Interval between sessions**	***N***	**Montage**	**Contact area of electrodes**	**Sham group**	**Results/insights**
Liebetanz et al., 2006b	Anticonvulsant effects of tDCS in the rat cortical ramp model of focal epilepsy	Original article	(1) c-tDCS (100 μA) for 30 and 60 min, anodal tDCS (100 μA) for 60 min, and 60 min of c-tDCS (*n* = 7) (2) c-tDCS (200 μA) for 15 and 30 min, anodal tDCS (200 μA) for 30 min, and c-tDCS for 30 min (*n* = 8)	(1) 4 sessions-−210 min (2) 4 sessions-−105 min	1 week	65 male Wistar rats	2 mm left and 2 mm anterior to the bregma	3.5 mm^3^	No	The anticonvulsive effect induced by c-tDCS depends on stimulation duration and current strength and may be associated with the induction of alterations of cortical excitability that outlast the actual stimulation.
Kamida et al., 2011	tDCS decreases convulsions and spatial memory deficits following SE in immature rats	Original article	Daily c-tDCS, for 30 min with an intensity of 200 μA, for 2 weeks (*n* = 6)	14 sessions-−420 min	24 h	18 male Wistar rats	1.5 mm to the right and 2 mm anterior to the bregma	3.5 mm^3^	No	Reduction of SE-induced hippocampal cell loss, supragranular and CA3 mossy fiber sprouting, and convulsions (reduction of 21%) in immature rats. tDCS treatment also rescued cognitive impairment following SE.
Kamida et al., 2013	c-tDCS affects SZs and cognition in fully amygdala-kindled rats	Original article	Daily 30 min c-tDCS with an intensity of 200 μA for 1 week (*n* = 6)	7 sessions-−210 min	24 h	18 male Wistar rats	1.5 mm to the right and 2 mm anterior to the bregma	3.5 mm^3^	Yes	c-tDCS treatment improved the SZ stage and decreased ADD together with elevated ADT 1 day after the last tDCS session. The treatment also yielded significant improvement in the performance of WMT. c-tDCS has anticonvulsive after-effects that last at least 1 day in the amygdale-kindled rats and positively affects cognitive performance.
Zobeiri and van Luijtelaar, 2013	Non-invasive tDCS in a genetic absence model	Original article	(1) 4 series of 15 min cathodal and anodal stimulation of 100 μA with an interval of 105 min in counter balanced order (*n* = 10) (2) 4 sessions of 15 min of cathodal stimulation of 100 μA (*n* = 8) (3) Similar protocol to (2), except that intensity was 150 μA (*n* = 8)	(1), (2) and (3) = 4 sessions-−60 min	105 min in all protocols	26 male WAG/Rij rats	Stimulation electrodes were placed on both hemispheres considering that the foci are bilateral. The two stimulation electrodes were fixed onto the cranium above the right and left somatosensory cortices (V/L −4.6 and V/L +4/6, respectively) and the reference electrode onto the cranium above the frontal cortex with no specific coordinate	3.5 mm^3^	No	c-tDCS reduced the number of SWDs during stimulation and affected the mean duration after stimulation both in an intensity-dependent manner. Behavior was changed after the highest stimulation intensity.
Dhamne et al., 2015	Acute SZ suppression by tDCS in rats	Original article	(1) Rats received tDCS sham tDCS, c-tDCS 1,000 μA, or c-tDCS 100 μA; for 20 min (*n* = 23, 22, 20, respectively) (2) 2 animal groups received a subtherapeutic lorazepam dose and then verum (c-tDCS 1000 μA) (*n* = 11) or sham tDCS (*n* = 10).	(1) and (2) = 1 session-−20 min		114 male Long Evans rats	Disk (active), and sponge (reference) electrodes were secured to the rat's scalp and torso, respectively.		Yes	Cathodal 1 mA tDCS reduced EEG spike bursts, and suppressed clinical SZs in combination with lorazepam and was more effective in SZ suppression and improved the clinical SZ outcomes compared to either tDCS or lorazepam alone.

*ADD: After-discharge duration; ADT: After-discharge threshold; c-tDCS: Cathodal transcranial direct-current stimulation; EEG: Electroencephalogram; SE: Status epilepticus; SWDs: Spike and slow-wave discharges; SZ: Seizure; tDCS: Transcranial direct-current stimulation; WMT: Water maze test*.

**Table 2:** Summary of tDCS and epilepsy clinical trials.

**Author (year)**	**Title**	**Type of article**	**Experiment**	**Total sessions**	**Interval between sessions**	***N***	**Montage**	**Contact area of electrodes**	**Sham group**	**Results/insights**
Fregni et al., 2006	A controlled clinical trial of cathodal DC polarization in patients with refractory epilepsy	A randomized, sham-controlled clinical trial	Single session of 1 mA c-tDCS for 20 min	1 session – 20 min		19 subjects (11 male and 8 female)	Cathodal electrode has been placed over the epileptogenic focus and the anode electrode over a silent area (without epileptogenic activity)	35 cm^2^	Yes (10 active and 9 sham)	c-tDCS reduced EDs (64.3%) and SZ frequency (44%) when compared with sham group (5.8%) and (11.1%) respectively.
Yook et al., 2011	Suppression of SZs by c-tDCS in an epileptic patient-a case report	Case report	5 days a week, during 2 weeks. Repeating procedure after 2 month, 20 min (2 mA for 20 min)	10 sessions-−200 min	24 h	1 subject (female)	Cathode electrode applied on midpoint between P4 and T4 area and anode electrode on left supraorbital area.	25 cm^2^		During the first 2 months after treatment; the patient had only six SZs, with an evident clinical improvement, after the second intervention the patient had just one SZ attack over 2 months.
San-Juan et al., 2011	tDCS in adolescent and adult Rasmussen's encephalitis	Case report	60 min in 4 sessions (on days 0, 7, 30, and 60) 1 mA for patient (1) and 2 mA for patient (2)	4 sessions-−240 min	7, 23 and 30 days respectively	2 subjects (male)	(1) (C3 [–/cathode]/contralateral supraorbital area [+/anode]) (2) (F2 [–/cathode]/F8 [+/anode])	Subdermal needle 12 mm in length and 0.4 mm in diameter		One patient was SZ free and another patient showed 50% SZ frequency reduction within 6 month of follow-up.
Faria et al., 2012	Feasibility of focal transcranial DC polarization with simultaneous EEG recording: preliminary assessment in healthy subjects and human epilepsy	Cross-over controlled trial with 15 healthy subjects and preliminary effects of its use, testing repeated tDCS sessions, in two patients with drug-refractory Continuous Spike-Wave Discharges During Slow Sleep (CSWS)	Once weekly, to 3 afternoon sessions of 30 min each. Current was ramped in steps of 0.1 mA, with a duration of 10 s each, until the target current of 1 mA.	3 sessions-−90 min	7 days	2 subjects (male)	Based in 10–10 International system positions in a cap (mostly C5-C6)	35 cm^2^		A large reduction after c-tDCS was found in IEDs in C5 (mean 32.1%) during and after tDCS (10 min).
Auvichayapat et al., 2013	tDCS for treatment of refractory childhood focal epilepsy	Controlled study	Single session of 1 mA c-tDCS for 20 min	1 session-−20 min		36 subjects (26 male and 10 female)	Cathodal electrode was placed over the epileptogenic focus, centered on the electrode with the international 10-20 EEG electrode placement system location where spikes of sharp waves were greatest in amplitude, and the anodal electrode was placed over the contralateral shoulder area.	35 cm^2^	Yes (27 active and 9 sham)	c-tDCS can suppress EDs frequency in 57.6% for 48 h, but the effect of a single session on EEG abnormalities was not sustained for 4 weeks. A statistical reduction in the frequency of SZs was found (4.8%) in the post-hoc analysis.
Assenza et al., 2014	Efficacy of c-tDCS in drug-resistant epilepsy: a proof of principle	Single blind and sham-controlled study	Two sessions, (1 sham and 1 real on the 8th and 22th days) 1 mA intensity applied for 9 min	1 real session-−9 min		2 subjects (male)	Cathodal electrode has been placed over the epileptogenic focus and the anode electrode over the contralateral homologous region	12.25 cm^2^		Patients showed a consistent reduction of the SZ frequency: about 70% for Patient 1 and about 50% for Patient 2.
Tekturk et al., 2016b	The effect of transcranial direct current stimulation on SZ frequency of patients with mesial temporal lobe epilepsy with hippocampal sclerosis	A randomized cross-over study	2 mA for 30 min on 3 consecutive days	3 real sessions-−90 min	24 h	12 subjects (6 male/6 female)	Active electrode placed over the pathologically affected HS side (temporal region, either T3 or T4 electrode place), which was determined by both concordant cranial MRI and ictal or interictal EEG findings, depending on the availability of the seizure records, and reference electrode over the contralateral supraorbital region	35 cm^2^		Ten patients showed a more than 50% decrease in their SZ frequency after c-tDCS. Six patients were SZ-free in the post c-tDCS period of 1 month.
Auvichayapat et al., 2016	Transcranial Direct Current Stimulation for Treatment of Childhood Pharmacoresistant Lennox-Gastaut Syndrome: a Pilot Study	A randomized, double-blind controlled trial	Five consecutive days of 2 mA c-tDCS for 20 min	5 sessions-−100 min	24 h	22 subjects (14 male and 8 female)	The stimulation site over the left M1, located based on the international electroencephalography (EEG) 10/20 electrode placement system. The reference electrode was placed over the right shoulder area.	35 cm^2^	Yes (15 active and 7 sham)	Participants assigned to the active tDCS condition reported significantly more pre- to post-treatment reductions in SZ frequency and epileptic discharges that were sustained for 3 weeks after treatment.
Tekturk et al., 2016a	Transcranial direct current stimulation improves SZ control in patients with Rasmussen's encephalitis	Descriptive study of a small case series	First cathodal, then anodal (2 mA for 30 min on 3 consecutive days for non-sham stimulations), and finally sham stimulation with 2-month intervals	3 sessions – 90 min	24 h	5 subjects (2 male/3 female)	Active electrodes placed over the mostly affected area and reference electrodes over the contralateral mastoid region	35 cm^2^		After cathodal stimulation, all but one patient had a greater than 50% decrease in SZs frequency. Two patients who received modulated c-tDCS had better results. The longest positive effect lasted for 1 month.
Zoghi et al., 2016	The effects of cathodal transcranial direct current stimulation in a patient with drug-resistant temporal lobe epilepsy (case study)	Case report	2 sessions of 1 mA c-tDCS (9–20–9 protocol) during a total of 18 min, with 20 min rest after the first 9 min	2 sessions-−18 min	20 min	1 subject (female)	The active electrode (cathode, 3 × 4 cm) was placed over the right temporal lobe, and the return electrode (anode, 5 × 7 cm) was placed over the left supraorbital area	Cathode, 12 cm^2^ and anode, 35 cm^2^		SZs reduced from 6-10 per day to 0–3 SZs per day. SZ frequency remained as low as 0–3 per day for 4 months, and then started to increase again.
Assenza et al., 2017	Cathodal transcranial direct current stimulation reduces seizure frequency in adults with drug-resistant temporal lobe epilepsy: a sham controlled study	A double-blind, randomized, sham-controlled, crossover, monocentric study	1 real session of 1 mA c-tDCS during 20 min	1 session-−20 min	30 days	10 subjects (male)	The cathode was placed over the epileptic focus, localized by means of EEG interictal and ictal activity, and the anode over the contralateral homologous region	35 cm^2^		c-tDCS reduced the percent weekly seizure frequency more than sham stimulation, without any change in interictal epileptiform activity
San-Juan et al., 2017	tDCS in Mesial Temporal Lobe Epilepsy and Hippocampal Sclerosis	A randomized, double-blinded, placebo-controlled, 3-arm parallel group (placebo, 30 min/2 mA daily sessions for 3 days, and 30 min/2 mA daily sessions for 5 days) clinical trial	2 mA for 30 min on 3 or 5 consecutive days of treatment	3 sessions-−90 min or 5 sessions-−150 min	24 h	28 subjects (16 male and 12 female)	The cathode was positioned over the most active IED area (defined as the zone [electrodes] with the highest discharge amplitude and/or frequency, located with the 10/20 system) as observed on the scalp EEG immediately before applying the tDCS. The anode electrode was placed over a silent supraorbital area (i.e., without epileptogenic activity) contralateral to the stimulated MTLE-HS side	35 cm^2^	Yes (20 active and 8 sham)	c-tDCS of 3 and 5 sessions decreased the frequency of SZs and IEDs (baseline vs. immediately post-tDCS).

*c-tDCS: Cathodal transcranial direct-current stimulation; EDs: Epileptiform discharges; EEG: Electroencephalogram; IEDs: Interictal epileptiform discharges; SZs: Seizures; tDCS: Transcranial direct-current stimulation*.

From Tables 1,2, we observed that most clinical studies showed significant results with respect to epileptiform activity, though only 11 studies (91.67%) had positive results in clinical outcomes such as seizure frequency reduction. For safety, none of them showed an increase in seizure frequency or moderate to severe adverse effects. On the other hand, two preclinical studies confirmed the initial hypothesis that cathodal-tDCS induces anticonvulsant effect.

### Preclinical studies that have been successfully translated to clinical studies

#### Reduction in seizure frequency by cathodal tDCS

The first study analyzed results of tDCS in rats subjected to a cortical ramp model of focal epilepsy. In this model, the anticonvulsive effect induced by cathodal-tDCS (c-tDCS) varied according to the duration of stimulation and strength of the current. In addition, the effect may be associated to modulation of cortical excitability that outlasts the actual stimulation (Liebetanz et al., 2006b). Kamida et al. (2013) reported similar results using amygdala-kindled rats; c-tDCS treatment significantly improved the seizure stage, decreased after-discharge duration, and elevated after-discharge threshold 1 day after the last tDCS session. Similar findings have also been reported in clinical studies by Fregni et al. (2006), Yook et al. (2011), San-Juan et al. (2011), Auvichayapat et al. (2013), Assenza et al. (2014), Tekturk et al. (2016a,b), Auvichayapat et al. (2016), Zoghi et al. (2016), Assenza et al. (2017), and San-Juan et al. (2017), conducted in a total of 138 patients.

### Preclinical studies that have failed to be translated to clinical studies

#### Status epilepticus

In 2011, Kamida et al. (2011) used a model of pilocarpine-induced status epilepticus (SE) in immature rats, and demonstrated a 21% reduction in convulsions on postnatal day 55. There are no studies evaluating the effects of tDCS in patients experiencing SE. However, there is a case report (that does not meet the inclusion criteria for this review) that has tested this approach (Grippe et al., 2015). Therefore, this design might be a good opportunity for a future trial.

#### Neuroprotective effects in the hippocampus

c-tDCS has been reported to exert neuroprotective effects in the immature rat hippocampus, reducing SE-induced hippocampal cell loss, as well as supragranular and CA3 sprouting (Kamida et al., 2011). This hippocampal impairment is caused by seizure-induced neuronal damage and synaptic reorganization, which starts soon after SE (Covolan and Mello, 2000). This type of a study is difficult to perform in a clinical setting; however, it would clearly be worthwhile to test, for example after brain injury that may lead to further impairment in cortical areas and result in epileptogenic foci (D'Ambrosio and Perucca, 2004).

#### Effects in absence epilepsy

Zobeiri and van Luijtelaar (2013) using a genetic absence model of epilepsy, showed that c-tDCS reduces slow-wave discharges (SWDs) in rats during stimulation and affects the mean duration of SWDs after stimulation, both in an intensity-dependent manner. Behavioral changes were also observed in response to the highest stimulation intensity. Spectral analysis of EEG during stimulation revealed an increase in sub-delta and delta frequency ranges, suggesting that cortical cells were hyperpolarized. These preclinical findings have not been replicated clinically, as no clinical study has evaluated tDCS in patients with absence epilepsy. This constitutes another opportunity for future studies.

#### Combination of tDCS with specific drugs to test synergistic effects

In an acute seizure induced by pentylenetetrazole, c-tDCS reduced EEG spike bursts, and suppressed clinical seizures in rats. c-tDCS, in combination with lorazepam, was more effective in SS compared with either tDCS or lorazepam alone, and prevented loss of motor cortex inhibition during paired-pulse transcranial magnetic stimulation (ppTMS) accompanied by pentylenetetrazole injection. This study provides evidence of the neural substrate of the antiepileptic effects of tDCS through ppTMS measures and demonstrates that c-tDCS enhances GABAergic intracortical inhibition mediated by GABA_A_ signaling. Further, c-tDCS prevented the loss of GABAergic ppTMS inhibition that is expected with PTZ-mediated GABA_A_ antagonism. This corroborates the hypothesis that tDCS may influence neurotransmitter levels and receptor function in humans (Medeiros et al., 2012). Thus, a combination of c-tDCS and GABAergic pharmacotherapy could be proposed, for example with benzodiazepine treatment (Dhamne et al., 2015).

### Clinical findings not replicated in preclinical studies

Epilepsy is characterized by multiple heterogeneous syndromes with various etiologies and symptoms, insufficiently addressed in current animal models despite the number of experimental options (Kandratavicius et al., 2014; Depaulis and Hamelin, 2015). The choice of appropriate protocol remains a challenge; most animal models used in epilepsy research are models of epileptic seizures rather than epilepsy *per se*, making differentiation subjective (Löscher, 2011). Thus, there are still limitations and shortcomings regarding models of refractory epilepsy and epilepsy because of hippocampal sclerosis, which often compromise the translational application of preclinical findings. Nonetheless, such clinical findings represent an opportunity to perform preclinical studies in an attempt to establish reproducible animal models and clarify the mechanisms involved in both pathology and treatment with tDCS.

#### Effects in rasmussen's encephalitis

Rasmussen's encephalitis is a rare and progressive inflammatory disease that reaches one cerebral hemisphere, and leads to intractable partial-onset seizures. Currently, the only effective treatment is hemispherectomy, but this procedure may cause irreversible neurological deficits. In a case report, San-Juan et al. (2011) reported that one patient with Rasmussen's encephalitis was seizure free and another showed a 50% reduction in seizure frequency within 6 months of follow-up after tDCS treatment. In a recent study, two patients with Rasmussen's encephalitis received modulated c-tDCS (2 mA for 30 min on 3 consecutive days) and demonstrated reduced seizure frequency following stimulation. One patient showed more than 50% reduction in seizure frequency, and the longest positive effect lasted for 1 month (Tekturk et al., 2016a). Thus, tDCS may be used to treat this pathology in order to avoid or delay surgical intervention (San-Juan et al., 2011).

In another study, patients with Lennox-Gastaut syndrome received pharmacological treatment for 5 consecutive days and 2 mA c-tDCS over the primary motor cortex (M1) for 20 min. This combination was more effective in reducing seizure frequency and epileptic discharges than pharmacological treatment alone. This reduction was sustained for 3 weeks after treatment (Auvichayapat et al., 2016).

#### Effects in drug resistant epilepsy

Approximately one-third of epilepsy patients develop drug resistance, and only 50% can take benefit from the surgical removal of an epileptic focus (Assenza et al., 2017). Although surgery is an option in cases of drug resistant epilepsy, many patients have no access to medical centers that perform respective epilepsy surgery. Furthermore, some patients may have a seizure focus located in eloquent cortex where resection is likely to cause deficit (Auvichayapat et al., 2013).

Many models of refractory epilepsy have been developed over the past 20 years, which use two approaches: (1) seizures or epilepsy models resistant to antiepileptic drugs (for example, 6-Hz psychomotor seizure model in mice) and (2) chronic epilepsy models, such as kindling. Kindling involves the application of repeated excitatory stimuli to induce partial seizures, followed by subsequent generalized seizures. This leads to increased seizure length and severity with continuous stimulation (Löscher, 2011). It is important highlight that we did not find preclinical studies relating the use of tDCS in refractory epilepsy, resulting in a lack of mechanistic and neurochemical clarifications. An alternative treatment is neuromodulation, which represents an attempt to improve the quality of life of patients with refractory epilepsy.

In 2006, Fregni et al. (2006) conducted a controlled study applying c-tDCS in 19 patients with refractory epilepsy; this unprecedented study investigated the electrographic and clinical response to c-tDCS in this epileptic condition. Patients underwent one session of c-tDCS (20 min, 1 mA) targeting the epileptogenic focus. The active stimulation did not induce seizures and was well-tolerated, and the treatment promoted a large reduction in number of epileptiform discharges (EDs) in the EEG and in the frequency of seizures. These parameters were measured and compared before (baseline), immediately after, and 15 and 30 days after either sham or active stimulation.

Yook et al. (2011) demonstrated in a case report of bilateral perisylvian syndrome that tDCS, when applied over the midpoint between P4 and T4, had a lasting effect over a 2-month period following treatment termination, decreasing the duration of each seizure episode. For 2 months after the second treatment session, only one seizure attack occurred, a considerable improvement over the eight seizure attacks per month prior to tDCS, when the patient was treated only with antiepileptic drugs.

The large reduction in interictal epileptiform EEG discharges in two subjects with drug-refractory continuous spike-wave discharges during slow sleep suggests that the simultaneous application of tDCS treatment and EEG recording allows the assessment of safety parameters during treatment. This methodology ensures that the stimulation is sufficiently focal and provides a detailed evaluation of epileptic activity changes induced by tDCS, representing an attractive outlook for epilepsy treatment (Faria et al., 2012).

Auvichayapat et al. (2013) showed that a single session of active tDCS treatment was associated with significant reductions in epileptic discharge frequency in children with refractory epilepsy immediately, 24, and 48 h after tDCS treatment. In addition, 4 weeks after treatment, a small decrease in seizure frequency was detected.

In focal resistant epilepsy, two patients received c-tDCS (constant current of 1 mA) during a real session in a single-blind, sham-controlled study, followed by 1 month of observation. During this period, the patients or caregivers provided a detailed seizure calendar (frequency per week at basal, post-sham and post-tDCS time points). These patients experienced reduction in seizure frequencies of ~70 and 50% (Assenza et al., 2014).

#### Effects in epilepsy due to hippocampal sclerosis

Animals subjected to kainate or pilocarpine-induced SE develop spontaneous seizures after a pre-epileptic period or seizure free, this can be due hippocampal injury, resulting in an animal model of mesial temporal lobe epilepsy (MTLE) with hippocampal sclerosis (Sloviter, 2008). Although MTLE is well-described in in clinical studies, with respect to electrophysiological and histological parameters, it remains partially reproduced in most rodent models (Depaulis and Hamelin, 2015). Even if there animal models described, remains some doubt about their reliability and extrapolation of their findings to the clinical setting, which represents a limitation in the conduction of preclinical studies using tDCS in models of MTLE due to hippocampal sclerosis.

In a randomized, placebo-controlled, double-blinded clinical trial with 3 sessions, 5 sessions and placebo stimulation, 3 and 5 sessions of c-tDCS stimulation decreased the frequency of seizures and interictal epileptiform discharge (immediately post-tDCS vs. baseline) in adults with MTLE and hippocampal sclerosis, compared to sham tDCS (San-Juan et al., 2017). It is known that MTLE with hippocampal sclerosis is a drug-resistant focal epilepsy syndrome. Another study showed that 83.33% of MTLE patients who received modulated c-tDCS (2 mA for 30 min on 3 consecutive days) showed more than 50% reduction in seizure frequency during a 1-month follow-up. Moreover, 50% these patients were seizure-free in the 1-month period post-tDCS (Tekturk et al., 2016b).

Recently, the case of a patient with drug-resistant temporal lobe epilepsy was reported. tDCS reduced seizure frequency from 6–10 per day to 0–3 per day. Seizure diaries revealed that seizure rates remained low (from 0 to 3 per day) for 4 months, and then began to increase (Zoghi et al., 2016). A recent database of published tDCS clinical trials, authored by Lefaucheur, presents a detailed list of studies assessing the clinical effect of tDCS, including in epileptic patients (Lefaucheur, 2016).

## Discussion

In this study, we performed a systematic review of clinical and preclinical studies using tDCS as a therapeutic approach in the treatment of epilepsy. Most studies presented here involved the use of c-tDCS, and several have investigated the effects of c-tDCS on spontaneous neural activity and evoked motor responses of the central and peripheral nervous system. These studies provide evidence that the effects of tDCS involve a non-synaptic mechanism of action, based on changes in neural membrane function (Ardolino et al., 2005).

tDCS has been applied in the treatment of epilepsy, spasticity, movement disorders, peripheral vascular disease, and certain psychiatric disorders (Raghavan et al., 2008). The acute effects of weak tDCS on ongoing epileptiform activity are well-established in animal models. However, the underlying mechanism by which prolonged tDCS modulates seizure initiation propensity and epileptogenesis remains unknown (Jackson et al., 2016). In addition, animal studies suggest that prolonged cathodal tDCS (c-tDCS) has anticonvulsant effects. On the other hand, anodal tDCS (a-tDCS) has contrary effects, decreasing the threshold for producing the seizure activity (evident in EEG), while behavioral changes are not observed (Hayashi et al., 1988; Liebetanz et al., 2006b). Nonetheless, Tekturk et al. attempted to prevent the generation and propagation of seizures by applying a-tDCS. This attempt was based on the hypothesis that even if c-tDCS decreases cortical excitability, a-tDCS increases the effects of inhibitory connections (Tekturk et al., 2016a). Therefore, they used c-tDCS targeting the epileptic foci and a-tDCS targeting the surrounding normal cortical tissue, an approach that was not effective in reducing the frequency of seizures.

Epilepsy is a pathology with the intrinsic characteristic of hypersynchronous brain activity. Therefore, epilepsy represents a model for abnormal hyperexcitatory plastic changes within cortical circuitry (San-Juan et al., 2017). The heterogeneity reported when using tDCS to treat refractory epilepsy may partly be attributed to the different etiology of that pathology. Accordingly, different approaches may be assessed in distinct types of epilepsy, due to the paucity of studies available. For example, in drug-resistant post-traumatic epilepsy patients, only one double-blinded randomized control trial has been published (Fregni et al., 2006). The causes of refractoriness of epilepsy to drugs and surgical treatment remain unknown. However, one possible explanation is the presence of neuronal damage affecting other brain areas besides the hippocampus (Petrovski et al., 2010; Zhang et al., 2017). A morphometric study using magnetic resonance imaging showed that neuronal damage in patients with temporal lobe epilepsy extends beyond the hippocampus, and affects regions that connect to the hippocampus functionally and anatomically (van Elst et al., 2000). This finding suggests the presence of a neural network injury which underlies the clinical manifestations in these patients (Andrade-Valença et al., 2008). More specifically, MTLE is commonly associated with hippocampal sclerosis (Andrade-Valença et al., 2008).

A previous review by our research group (Medeiros et al., 2012) discusses the presumed mechanisms of action of tDCS, attempting to elucidate the underlying neurobiology and cell-signaling pathways involved. There, we suggest that tDCS induces plasticity, improves neuronal viability and morphology, modulates synaptic transmission, and biosynthesis of molecules.

tDCS has consistently been reported to be safe, and a recent review confirmed the absence of evidence for serious adverse effects (Bikson et al., 2016). tDCS is a technique that can be applied with low risk and little discomfort, and when used in repeated sessions, can have long-lasting effects (Nitsche et al., 2008). The effects of tDCS in the short term occur due to a decrease (anodal) or increase (cathodal) in neuronal firing threshold (Ruscheweyh et al., 2011). However, long-term effects involve the participation of brain-derived neuronal factor (BDNF) and glutamatergic N-methyl-d-aspartate (NMDA) receptors in synaptic plasticity mechanisms (Fertonani et al., 2010). Brain damage induced by the formation of toxic products does not occur using this technique, because there is no direct contact of electrodes with the cerebral cortex (Nitsche et al., 2003). Magnetic resonance imaging before and after 30 and 60 min of stimulation applied to the prefrontal and motor cortex did not exhibit pathological signal alterations. As such, it was concluded that tDCS does not induce cerebral edema, or render abnormal the blood brain barrier or brain tissue (Rosen et al., 2009). Finally, Accornero et al. (2007) showed no abnormal variations in heart rate, blood pressure, or temperature during and 20 min after the end of the stimulation. Therefore, tDCS is a safe method for use in humans, and has the advantage of being easily combined with other interventions, as pharmacological treatment.

San-Juan et al. (2015) reviewed the efficacy and safety of tDCS in epilepsy. The authors analyzed 9 articles using different methodologies (3 pre-clinical/6 clinical). Moreover, *in vivo* and *in vitro* animal studies demonstrated that direct current stimulation could induce suppression of epileptiform activity without neurological injury. Four out of six (67%) clinical studies revealed an effective decrease in epileptic seizures, and five out of six (83%) showed a reduction of interictal epileptiform activity (San-Juan et al., 2015; Scorza and Brunoni, 2015). In fact, in this review we did not find evidence that tDCS in epilepsy may lead to an increase in seizures or any other significant adverse effects (Pereira et al., 2016). Additional studies involving a large cohort of patients are required to investigate the effects of tDCS in drug-resistant epilepsy.

**Figure 4:**
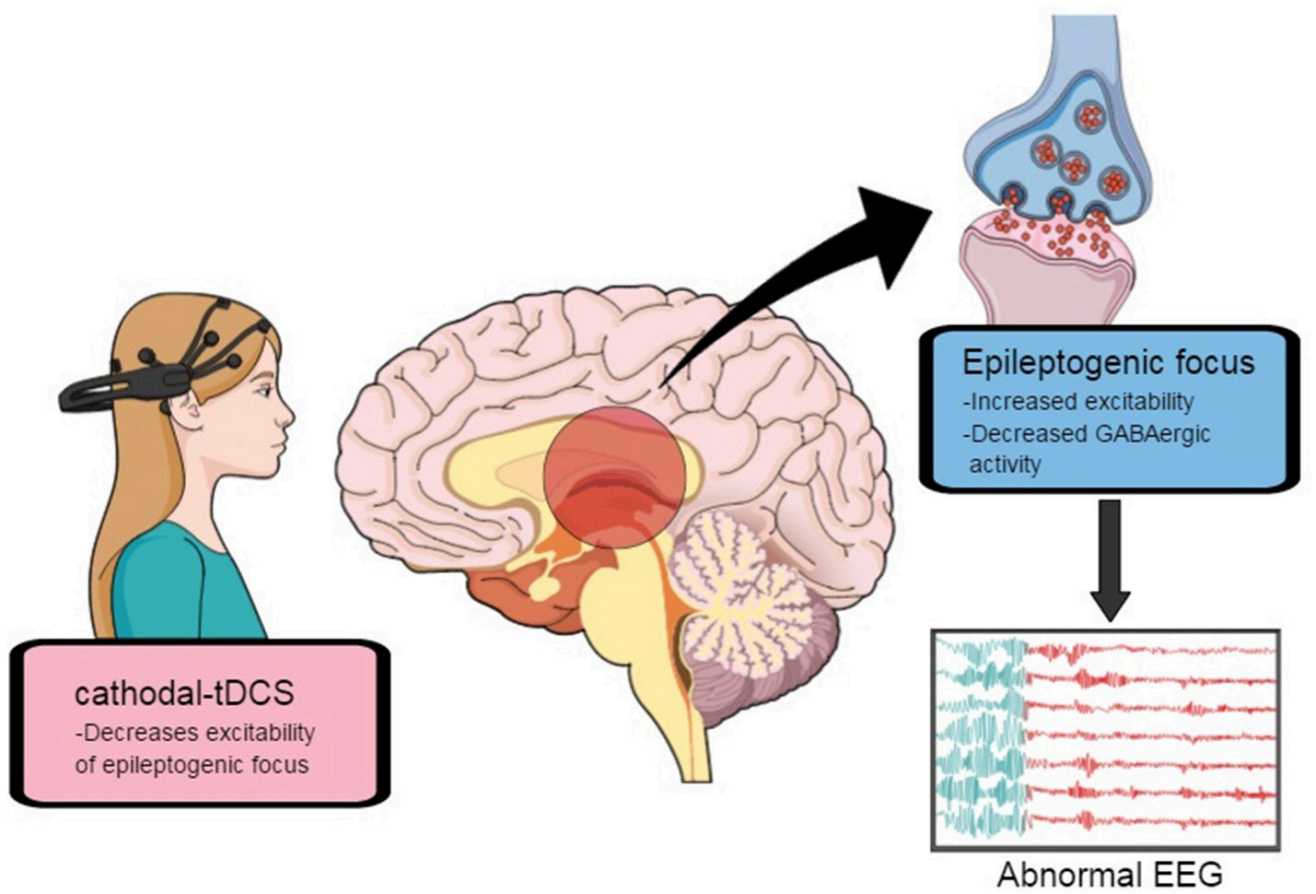
Excitability in epileptogenic focus might be decreased by cathodal transcranial direct-current stimulation. Adapted from Fregni and Pascual-Leone (2007).

In order to develop optimal stimulation protocols and long-term follow-up, animal studies and larger prospective clinical trials with homogenous epileptic conditions are needed. Every study uses different patient categories, stimulation protocols, electrode sizes, stimulation sites, and stimulation current strength. Therefore, conclusions drawn from the comparison of these studies should be used to provide standardized measures, in order to improve reproducibility of outcomes (Gschwind and van Mierlo, 2016). Epileptogenesis involves an increase in excitatory synaptic strength, and seizure foci are characterized by a pathological reduction of inhibitory (GABA-releasing) terminals and an increase in excitatory (glutamatergic) terminals (Figure 4; Fregni and Pascual-Leone, 2007). Hence, the principle mechanism of action of tDCS might be the induction of long-term-depression-like (LTD) effects, i.e., reducing cortical excitability and the probability of paroxysmal activity in epileptogenic cortical regions (Nitsche and Paulus, 2009). While the immediate anticonvulsant effects of c-tDCS involve the hyperpolarization of neuronal soma and desynchronization of neuronal activity, its long-term effects seem to occur through the modulation of synaptic transmission, causing LTD in the thalamus-cingulate pathway. This process appears to be N-methyl-D aspartate (NMDA) receptor- and duration-dependent (Chang et al., 2015), thus c-tDCS seems to promote intracortical inhibition. On the other hand, a-tDCS facilitates synaptic plasticity mediated by a long-term potentiation (LTP)-like mechanism, as well as previous studies presented that brief seizures could induce LTP and mossy fiber sprouting in the hippocampus; therefore, the mechanism of LTP formation might be similar to the mechanism of epileptogenesis (Chang et al., 2015; Rroji et al., 2015). Finally, the mechanisms underlying the effects of tDCS seem to be involved not only in local polarity-related modifications of cortical excitability, but also in more complex interhemispheric connections (Tatti et al., 2016).

## Conclusions

Considering the data obtained in this review, we conclude that tDCS should be considered a viable therapeutic option in refractory epilepsy, particularly in patients who are unable to undergo surgery. In general, animal studies used c-tDCS with currents ranging from 100 to 200 μA; even with defined montages, the stimulation appears to be bicephalic, due to the animal's skull size. At present, clinical studies involving c-tDCS use ranging from 1 to 2 mA, and the cathode is placed over the epileptic foci in majority. Cathodal tDCS appears to decrease excitability through hyperpolarization associated to LTD-like mechanisms. Moreover, because tDCS is simple to use, low cost, and easily accessible, it is a good option in countries with limited resources (Scorza and Brunoni, 2015; Zoghi et al., 2016). Therefore, despite the intriguing possibility of modulating neural networks, tDCS still requires a more in-depth analysis of the most beneficial protocols and elucidation of the underlying mechanism of action. Thus, this non-invasive technique still requires further sham-controlled, double-blind larger multi-center studies, which may be justified for cost-effectiveness and surgery complication avoidance. Novel methods of real-time assessment with EEG, and use of other neural markers, may also help with understanding the clinical effects of tDCS in epilepsy (Faria et al., 2012; Leite et al., 2017). Although there are several trials published in this field, further evidence is needed to understand the potential role of using tDCS to treat epilepsy.

## Author contributions

All authors participated in the design of the study and drafted the manuscript. IT and PP participated in study coordination and helped draft the manuscript. FF and DL helped finalize the manuscript. GR, CdO, and RV have designed and prepared the manuscript figures. All authors read and approved the final manuscript.

### Conflict of interest statement

The authors declare that the research was conducted in the absence of any commercial or financial relationships that could be construed as a potential conflict of interest.
